# The Role of fMRI to Assess Plasticity of the Motor System in MS

**DOI:** 10.3389/fneur.2015.00055

**Published:** 2015-03-16

**Authors:** Patrizia Pantano, Nikolaos Petsas, Francesca Tona, Emilia Sbardella

**Affiliations:** ^1^Department of Neurology and Psychiatry, Sapienza University of Rome, Rome, Italy; ^2^IRCCS Neuromed, Pozzilli, Italy

**Keywords:** fMRI, functional connectivity, motor system, multiple sclerosis, neuroplasticity, resting-state fMRI

Neuroplasticity in the motor system has been extensively investigated by functional MRI (fMRI) in multiple sclerosis (MS) patients; we report results obtained by both task-related and resting-state fMRI studies. Furthermore, the possibility to manipulate neuroplasticity in MS will also be addressed.

## Task-Related fMRI Studies

The first works on fMRI and the motor system reported greater cortical activation in patients with relapsing–remitting (RR) or secondary progressive (SP) MS than in healthy subjects (HS) during a simple finger flexo-extension hand movement (1, 2). This increased activity also involved the ipsilateral hemisphere, especially in patients with more severe axonal damage (2). Increased cortical activity during the performance of the same simple motor task was also observed in patients with primary progressive MS (3). In the same years, we described a large increase in motor activation, with a greater involvement of the ipsilateral hemisphere, in MS patients following a first clinical episode of motor deficit from which they had fully recovered (4). In a subsequent study, we assessed cortical activity during the same thumb-to-finger opposition task in patients with a clinically isolated syndrome (CIS) after clinical recovery, divided into an optic neuritis group and a paresis group (5), with the aim to better investigate patterns of motor reorganization. A greater involvement of the ipsilateral hemisphere in the paresis group, not only versus HS but also versus the optic neuritis group, suggested that neuroplastic changes in the motor system contribute to the recovery and maintenance of a normal motor function level, despite the presence of structural damage.

Functional MRI activation during a motor task should be viewed as a dynamic phenomenon that changes during the disease course. In a single-case study, Reddy and collaborators reported a progressive reduction in cortical over-activation paralleling motor improvement after a clinical relapse and NAA recovery at spectroscopy (6). We longitudinally evaluated 18 patients with RRMS by performing two fMRI studies in the remitting phase, on average 20 months apart (7). Decreased ipsilateral motor activation, which inversely correlated with age, progression of T1 lesions and occurrence of new relapses, was observed at follow-up. In other words, in patients with a less severe disease course, motor activation tended to return to a more normal pattern. In keeping with our findings, Mezzapesa et al. reported that pseudo-tumoral MS lesions affecting the motor system lead to the recruitment of pathways in the ipsilateral hemisphere; good recovery after relapses is associated with function recovery in the contralateral motor areas and decreased ipsilateral activation (8).

New insights come from task-related fMRI studies in different MS phenotypes. In a cross-sectional study, Rocca et al. evaluated patients with CIS, RRMS, or SPMS (9). They found various patterns of motor activation, which spread as the phenotype became clinically more severe: a more lateralized pattern in CIS, a more bilateral pattern in RRMS, and the recruitment of additional areas, even outside the motor system, in SPMS. Therefore, over-activation does not necessarily represent adaptive plasticity since it may even be associated with a high disability, as observed in SPMS; it is conceivable that over-activation, to some extent, limits the clinical manifestations of tissue damage, without fully compensating.

The MS widespread microstructural damage, as shown in combined diffusion tensor imaging (DTI) and fMRI studies, correlates with increased sensorimotor network activation (10, 11). A strict correlation between over-activation of the motor areas and structural damage specifically located along the cortico-spinal tract has been documented, suggesting a compensatory role (4, 12). On the other hand, over-activation in the ipsilateral motor cortex significantly correlated with callosal damage (11, 13), suggesting that increased activity in the ipsilateral motor cortex is likely due to decreased inhibitory input of trans-callosal fibers and thus represents a marker of disease severity rather than a mechanism of adaptive plasticity.

Multiple sclerosis patients display greater cortical activation than HS even during passive movements of a limb, which, unlike active movements, are not affected by individual motor impairment (6, 14). In agreement with the data yielded by active movements (9), passive movements of the hand induced a progressive extension of activation to the ipsilateral hemisphere according to the clinical phenotype (HS < RRMS < SPMS) (15). Deactivation of posterior cortical areas belonging to the default mode network (DMN) increased in RRMS, though not in SPMS, if compared with HS; activation in the contralateral sensorimotor cortex was significantly correlated with deactivation in the DMN in HS and RRMS, though not in SPMS. These findings suggest that disorganization between anti-correlated functional networks is due to a higher level of disconnection.

## Resting-State fMRI Studies

Recent years have witnessed a growing interest in the study of resting-state functional connectivity (rs-FC) in MS aimed at understanding alterations in the intrinsic functional architecture of the MS brain and their role in disease progression and clinical impairment. Resting-state fMRI (rs-fMRI) can be used to identify anatomically separate, though functionally connected, brain regions configuring specific RS networks (16, 17); unlike fMRI during movement execution, rs-fMRI is not influenced by task performance, which may differ from that of HS, especially in patients with motor disability.

Some studies have reported a reduced rs-FC in the sensorimotor network in MS. Lowe et al. demonstrated a bilaterally reduced rs-FC in the motor cortices in patients with varying degrees of MS, both in the resting state and during finger tapping, thereby showing that both these fMRI approaches differentiate patients with MS from controls (18). In a large group of RRMS patients with a wide range of disabilities and disease durations, Rocca et al. found decreased rs-FC in regions of the sensorimotor network in RRMS when compared with HS; moreover, the authors hypothesized a link between the reduction in rs-FC and severity of tissue damage (19).

In contrast, other studies have reported increased rs-FC in the motor network in early MS (20) and in RRMS patients with mild disability (21); they suggested that the rs-FC increase is an early phenomenon of cortical reorganization that is lost as the disease progresses. A recent multi-center study revealed a significant generalized increase in rs-FC within the sensorimotor network in a heterogeneous group of MS patients, which once again points to a potential role of this rs-FC widespread enhancement in maintaining brain functionality (22).

Several factors may explain the discrepancies between studies reporting decreased or increased rs-FC in MS, including differences in the patients’ clinical characteristics (e.g., in clinical subtype, disease duration, and clinical disability), number of subjects enrolled, and methods used for both image acquisition and analysis. However, when the findings of these studies are considered together, they point to a functional reorganization of the motor network in MS patients, which is present from the earliest disease stages.

More recently, some studies attempted to explore the clinical correlate of motor rs-FC alterations in MS. Indeed, although the ability of rs-fMRI to detect brain functional reorganization in MS has been proved, the role of FC alterations in the pathogenesis of MS, as well as the potential relationship between resting-state network reorganization and clinical disability, remain unclear.

In a recent work, Janssen and collaborators demonstrated reduced intra-network connectivity in the motor network in RRMS patients, associated with higher levels of disease severity, thus pointing to the possibility that resting-state changes may serve as a biomarker of disease progression (23). On the other hand, increased connectivity in the left premotor area was found to be associated with greater clinical disability in RRMS though not in SPMS (24). This finding suggests that even if disease progression is related to disrupted FC within the motor network, increased FC in specific motor areas may represent an attempt to compensate for the functional impairment, at least in RRMS.

## Modulation of Neuroplasticity

The objectives of neuroimaging studies should be to distinguish between beneficial and non-beneficial (maladaptive) neuroplastic changes and to understand whether, and if so how, we can modulate brain plasticity to enhance cortical activity changes associated with a clinical improvement. Studies on the effects of drugs or motor practice on cortical activity are particularly interesting in this regard (25–29).

In a double-blind, crossover, placebo-controlled study, we evaluated the short-term effect of a single dose of 3,4-dyaminopyridine (DAP), a K-channel blocker shown to improve motor function and fatigue, in RRMS patients with mild disability (26). fMRI during a right-hand movement demonstrated greater activation in the right motor areas after 3,4 DAP compared with placebo, which was instead associated with a subjective improvement in fatigue. Similarly, TMS led to reduced intracortical inhibition and increased intracortical excitation after 3,4 DAP compared with placebo. We therefore concluded that this drug might improve motor function by enhancing excitatory synapses.

The effects of a short motor training in MS patients have been reported in two task-related fMRI studies, though with discrepant results (28, 29). Morgen et al. showed that MS patients did not display any decrease in motor activation in the contralateral primary motor and parietal cortices after motor training, which in HS is interpreted as adaptation to a simple, automated movement. Mancini et al. instead showed that motor training induces a progressive decrease in cerebral activation in sensorimotor system areas in both HS and MS patients, thereby suggesting that the physiological process of short-term adaptation to a simple motor training is preserved in MS.

Tomassini et al. showed that both short- and long-term (15 days) visuo-motor practice induced the same level of improvement in HS and patients (25). Moreover, their fMRI study revealed changes in activated areas, which, however, differed between patients and HS. Their results suggest that neuroplasticity induced by visuo-motor practice is preserved in MS, although underlying mechanisms differ from those in healthy people.

This conclusion is supported by our recent work on FC changes in early RRMS patients (27), who were studied by rs-fMRI before and after a short motor training, i.e., a 25-min repetitive thumb flexion with the right hand, that closely resembled that described in previous fMRI studies (24, 25). The study of the sensorimotor (SMN) and cerebellar (CBN) networks revealed no pre-training rs-FC differences between MS patients and HS; differences did instead become manifest after motor practice. The SMN displayed post-training FC increase in both groups, which, however, reached statistical significance only in HS, whereas the CBN FC significantly increased in RRMS alone. Interestingly, following motor training, a significant correlation was observed in patients between the rs-FC of the SMN and CBN, suggesting an emerging inter-network synchronization. Furthermore, the FC increase in the SMN significantly correlated with tissue damage, as assessed by lesion volume and fractional anisotropy. The manipulation of the resting-state to define its dynamics might be a valid way to investigate functional connectivity alterations in patients.

## Conclusion

Functional MRI studies exploring the motor system in MS have demonstrated the ability of the brain to reorganize itself as a response to the disease. Functional reorganization develops in relationship with structural disconnection, making the structural substrate evaluation essential. Despite the undeniable progress in fMRI techniques, clinical interpretation is still controversial and no single technique has proved adequate to predict clinical evolution, ultimately because of the knowledge gap between brain connectivity and function.

Future studies integrating rest and task fMRI (30) might allow us to obtain the “best of both worlds” by shedding light on altered interactions between those two brain function states. Within the context of RS–FC characterization, there is growing interest in the analysis of the intrinsic dynamics of RS time courses and spatial maps (31). This type of assessment (32) has already revealed alterations in DMN dynamics in early MS subjects. Network analysis tools, based on inter-network correlations and graph theoretical analysis, are also very promising (33). Task-manipulated resting-state to elicit altered responses in MS opens perspectives for assessing targeted functions. The nature of changes observed in fMRI will be established in the measure of our further knowledge on brain’s dynamics, under task and/or in resting-state; the ambition is to reveal every patient’s potential for experience-dependent plasticity, thus pinpointing a target for neurorehabilitation and identifying successful intervention markers.

## Conflict of Interest Statement

The authors declare that the research was conducted in the absence of any commercial or financial relationships that could be construed as a potential conflict of interest.
